# Molecular Detection of Mutations in the *penA* and 23S rRNA Genes of *Neisseria gonorrhoeae* Related to Decreased Cephalosporin and Azithromycin Susceptibility in Rectal Specimens from Men Who Have Sex with Men (MSM) in Lima, Peru

**DOI:** 10.3390/tropicalmed10080211

**Published:** 2025-07-28

**Authors:** Francesca Vasquez, Maria Eguiluz, Silver K. Vargas, Jazmin Qquellon, Carlos F. Caceres, Jeffrey D. Klausner, Kelika A. Konda

**Affiliations:** 1 Centro de Investigación Interdisciplinaria en Sexualidad, SIDA y Sociedad, Universidad Peruana Cayetano Heredia, Lima 15074, Peru; maria.eguiluz.m@upch.pe (M.E.); silver.vargas.r@upch.pe (S.K.V.); luz.qquellon@upch.pe (J.Q.); carlos.caceres@upch.pe (C.F.C.); kelikakonda@gmail.com (K.A.K.); 2 Department of Population and Public Health Sciences, Keck School of Medicine, University of Southern California, Los Angeles, CA 90089, USA; jdklausner@med.usc.edu

**Keywords:** *Neisseria gonorrhoeae*, *penA*, 23S rRNA, antimicrobial resistance, qPCR

## Abstract

*Neisseria gonorrhoeae*, the causative agent of gonorrhea, represents a major public health concern due to its increasing antimicrobial resistance. While often asymptomatic—particularly in extragenital infections—untreated cases can lead to severe complications and further transmission. Despite global efforts to monitor antimicrobial resistance, data on the molecular determinants underlying decreased susceptibility in *N. gonorrhoeae* remain scarce in Peru. This study aimed to detect mutations in the *penA* and 23S rRNA genes, which confer decreased susceptibility to cephalosporins and azithromycin resistance. We extracted DNA from 124 *N. gonorrhoeae*-positive clinical rectal specimens collected in Aptima Combo 2 transport tubes from MSM patients. These DNA samples were then screened using the Mismatch Amplification Mutation Assay-based real-time PCR (MAMA-qPCR) to identify mutations in the 23S rRNA and *penA* genes. Each sample underwent separate reactions to detect A2059G and C2611T mutations in the 23S rRNA gene, and 86 of these samples were further tested in individual qPCR assays for the *penA* D345 deletion (D345del) or G545S mutations. Sanger sequencing was performed on all DNA samples positive for 23S rRNA mutations by MAMA-qPCR assay, and on 27 DNA samples that yielded sufficient *penA* amplicons for additional sequencing. Using the MAMA-qPCR assay for the 23S rRNA gene, 64 of 124 samples amplified in the A2059G reaction: 2 (3.1%) carried the mutation, and 62 were classified as wild type. In the C2611T reaction, 42 of 124 samples amplified, and none of them carried the mutation. Using the MAMA-qPCR assay for the *penA* gene, we only analyzed 86 samples, as the remaining 38 samples had insufficient DNA yield. A total of 44 of the 86 samples amplified in the D345del reaction: 5 (11.4%) carried the D345del, and 39 were classified as wild type. In the G545S reaction, 4 (6.4%) carried the mutation, and 58 were classified as wild type. Finally, sequencing of the *penA* gene in the 27 samples revealed mutations related to decreased susceptibility to cephalosporins. This study identified genetic mutations conferring resistance to azithromycin and decreased susceptibility to cephalosporins, providing an overview of the circulating mutations conferring resistance in *N. gonorrhoeae* strains in Peru.

## 1. Introduction

*Neisseria gonorrhoeae* is the second most prevalent bacterial sexually transmitted infection (STI), with an estimated annual global incidence of 82.4 million cases [1]. High incidence and prevalence are commonly observed in key populations such as men who have sex with men (MSM) and transgender women (TW), and this infection is associated with a higher risk for HIV acquisition [2,3,4]. Asymptomatic extragenital infections are common among key populations [5,6]. In Peru, a previous study among MSM and TW yielded a 9.6% prevalence of anal *N. gonorrhoeae* infections [7]. This convergence of high prevalence in key populations, asymptomatic transmission, and rising antimicrobial resistance underscores the critical need for molecular surveillance.

Antimicrobial resistance (AMR) represents a major concern for managing *N. gonorrhoeae* infections [8,9]. Although the World Health Organization (WHO) currently recommends dual therapy with a third-generation cephalosporin (ceftriaxone or cefixime) plus azithromycin as a reliable treatment option, the global spread of *N. gonorrhoeae* resistant strains has been reported worldwide, yielding untreatable infections [1,10,11,12,13]. Multidrug-resistant *N. gonorrhoeae* imposes severe socioeconomic burdens through prolonged infections, infertility, and treatment costs [1].

Few studies on *N. gonorrhoeae* resistance have been conducted in Peru. A previous study performed on culture isolates from male patients with *N. gonorrhoeae* infection showed a resistance rate of 9.1% to azithromycin and decreased susceptibility to ceftriaxone in 1.2% and cefixime in 3.6% of the isolates [14]. In addition, in a study from our group in Peru, performed directly on *N. gonorrhoeae*-positive rectal swab specimens using qPCR, we found that 84% of samples carried the S91F mutation in the *gyrA* gene, which is known to confer resistance to ciprofloxacin [15].

Azithromycin-resistant *N. gonorrhoeae* strains have alterations at the 2611 and 2059 positions (*Escherichia coli* numbering) in the peptidyltransferase loop in domain V of the 23S rRNA gene. The mutation C2611T in the 23S rRNA gene has been highly associated with low to moderate-level azithromycin resistance. Additionally, the A2059G mutation is associated with high-level azithromycin resistance, defined as resistant strains with azithromycin minimum inhibitory concentrations (MICs) in vitro ≥256 mg/L [16,17,18].

Additionally, decreased susceptibility to cephalosporins in *N. gonorrhoeae* strains has been reported worldwide [19,20,21,22]. Mutations in the *penA* gene are widely recognized as the main mechanism through which cephalosporins resistance is acquired through genetic alterations in mosaic and non-mosaic *penA* alleles [23]. The insertion of an extra aspartate in the codon 345 (D345ins) may affect the active site of the penicillin binding protein 2 (PBP-2) encoded by the *penA* gene, resulting in a conformational change, leading to decreased affinity for specific antimicrobials [24]. Additional alterations on the *penA* gene have been extensively studied; the presence of any mutated codon at positions 375–377, 501, or 551 of the *penA* gene accurately predicts decreased susceptibility to cefixime [22,25].

Current research on *N. gonorrhoeae* resistance focuses on the development of Nucleic Acid Amplification Tests (NAATs) aimed at detecting significant mutations associated with resistant phenotypes, directly from clinical samples [15,26]. This would bypass the need for culture, which has lower detection rates [27]. This study aimed to detect mutations in the *penA* and 23S rRNA genes highly associated with decreased susceptibility to cephalosporins and azithromycin resistance from rectal *N. gonorrhoeae* samples. The findings from this study will contribute to the review and update of the treatment guidelines for *N. gonorrhoeae* and assist in decision-making regarding the surveillance, management, and control of molecular antimicrobial resistance at the national and global levels.

## 2. Materials and Methods

### 2.1. Screening of Rectal Clinical Samples Using Aptima Combo 2 Assay

A total of 124 Aptima-positive rectal clinical specimens were analyzed. One set of 65 samples was collected between 2013 and 2016, as part of the PICASSO cohort [28], obtained from MSM and TW participants at high risk of acquiring syphilis infection in Lima, Peru. The remaining 59 samples were collected between 2019 and 2021, as part of the Prospero study [29], from MSM participants attending STI clinics in Lima, Peru. All rectal clinical specimens were self-collected using Aptima Combo 2 CT/NG (AC2) transport buffer kit (Hologic Inc., San Diego, CA, USA). The residual buffer of the Aptima C2-positive samples was stored at −80 °C until DNA extraction.

### 2.2. DNA Extraction and Real-Time PCR Assay

We extracted DNA from 124 rectal clinical specimens positive for *N. gonorrhoeae* (Aptima C2) using QIAamp DNA minikit (Qiagen, Mississauga, ON, Canada), following the manufacturer’s protocols. Subsequently, we performed a Mismatch Amplification Mutation Assay-based real-time PCR (MAMA-qPCR) to identify two-point mutations in each *penA* and 23S rRNA genes using specific primers described in Table 1. The MAMA-qPCR assay originally developed by Donà et al. is an SYBR-green-based real-time PCR method that uses mutation-specific primers to amplify and identify mutant *N. gonorrhoeae* strains directly from clinical specimens, thereby predicting antimicrobial resistance without requiring culture [26].

We processed all 124 *N. gonorrhoeae*-positive DNA samples to detect resistance-associated mutations in the 23S rRNA gene by MAMA-qPCR assay. Each DNA sample underwent two independent qPCR reactions: one employing the primer set specific for the A2059G mutation, related to high-level azithromycin resistance, and another using the primer set targeting the C2611T mutation, related to low to moderate-level resistance.

For the detection of *penA* mosaic alleles, 86 of the 124 DNA samples were analyzed; the remaining 38 DNA samples could not be processed due to insufficient DNA yield. Each DNA sample underwent two independent qPCR reactions: one using the primer set targeting the D345del mutation, and another employing the primer set targeting the G545S mutation.

The PCR cycling protocol started with a denaturation step at 95 °C for 10 min; 40 cycles of denaturation at 95 °C for 15 s, annealing at 62 °C for 10 s, and extension at 72 °C for 10 s. All rectal clinical specimens were processed in duplicate using a cycle threshold (C_T_) < 40 cycles [15,26].

Two clinical isolates of *N. gonorrhoeae* with well-characterized azithromycin phenotypes were included: one showing low to moderate-level resistance and carrying the C2611T mutation, and another exhibiting high-level resistance with the A2059G mutation. Additional clinical isolates with decreased susceptibility to cephalosporins, each carrying a specific *penA* mutation such as D345del or G545S, were also used, while fully susceptible isolates lacking these mutations in the 23S rRNA or *penA* genes were used as negative controls. All isolates were provided by Dr. Klausner’s laboratory (USA).

### 2.3. Sanger Sequencing

To confirm whether the 23S rRNA mutation detected by MAMA-qPCR was present in all four alleles, we applied a two-step PCR followed by Sanger sequencing [16]. First, each allele was amplified with the forward primer gonrRNA-F and four reverse rRNA_1 to rRNA_4 primers listed in Table 1. The PCR cycling protocol started with a denaturation step at 95 °C for 10 min; 30 cycles of denaturation at 94 °C for 1 min, annealing at 66 °C (alleles 2 and 3) or 68 °C (alleles 1 and 4) for 1.5 min, and extension at 72 °C for 2.5 min. The resulting products served as templates for a second PCR with gonrRNA-F and gonrRNA-R2 (reverse) primers; the PCR conditions were 35 cycles of denaturation at 94 °C for 1 min, annealing at 59 °C for 1 min, and extension at 72 °C for 1 min. Sanger sequencing was performed using the ABI 3730xl DNA Analyzer (Applied Biosystems, Foster City, CA, USA) platform. The resulting electropherograms were analyzed, and sequences were aligned using multiple sequence alignment by the EMBL-EBI Job Dispatcher sequence analysis tool (https://www.ebi.ac.uk/jdispatcher, accessed on 10 January 2025).

An additional conventional PCR was also performed on all 124 DNA samples to enable subsequent *penA* sequencing, using previously reported PenA-A3 (forward) and PenA-B3 (reverse) primers [30]; however, only 27 samples amplified. The PCR cycling protocol started with a denaturation step at 95 °C for 10 min, followed by 35 cycles of denaturation at 94 °C for 1 min, annealing at 62 °C for 1.5 min, and extension at 72 °C for 2.5 min. Primer sequences and targets are detailed in Table 1. Then, the final third portion of the *penA* gene on these 27 samples was sequenced by the Sanger method. The presence of any amino acid variations in the PBP-2 (encoded by the *penA* gene) was investigated by translating the nucleotide sequences obtained using the BLASTx online program. The translated amino acid sequences were then compared to the wild-type *N. gonorrhoeae* strain (GenBank accession no. M32091) to evaluate the gene codons associated with decreased susceptibility to cefixime. The resulting electropherograms were analyzed, and sequences were aligned using multiple sequence alignment by the EMBL-EBI Job Dispatcher sequence analysis tool (https://www.ebi.ac.uk/jdispatcher). Accessed on 10 January 2025.

### 2.4. Statistical Analysis

Data analysis was limited to descriptive statistics (frequencies and proportions) using STATA v18.0 (College Station, TX, USA), consistent with the study’s surveillance objectives.

## 3. Results

### 3.1. Detection of Mutations in the 23S rRNA and penA Genes by MAMA-qPCR

For detecting the A2059G mutation in the 23S rRNA gene, 64 out of 124 DNA samples were amplified. Among these, 62 samples were classified as wild type, while two samples (3.1%) were classified as mutants carrying the A2059G mutation. Both mutant samples were collected during 2020–2021. For the C2611T mutation in the 23S rRNA gene, 42 out of 124 samples were amplified, and all of them were classified as wild type.

For detecting the D345del in the *penA* gene, 44 out of 86 DNA samples were amplified. Among these, 5 samples (11.4%) carried the D345del mutation and were classified as mosaic type alleles, while 39 samples were classified as wild type. For detecting the G545S mutation, 62 out of 86 DNA samples were amplified. Among these, 4 samples (6.4%) were classified as mutants, carrying the G545S mutation, while 58 samples were classified as wild type. A complete summary of all mutations detected by MAMA-qPCR in the 23S rRNA and *penA* genes is provided in Table 2.

### 3.2. Analysis of Mutations in the 23S rRNA and penA Genes by Sanger Sequencing

The A2059G mutation was previously detected in two samples using the MAMA-qPCR assay; however, only one sample was sequenced by Sanger, as the other sample failed to amplify through conventional PCR, which precluded it from further processing. In the sequenced sample, the A2059G mutation was found in the four 23s rRNA alleles (4/4), validating the MAMA-qPCR result. Additionally, neither of the two samples with this A2059G mutation exhibited the D345del or G545S mutations in the *penA* gene at the same time.

Mosaic *penA* mutations were also detected at codons 345 and 545 using the MAMA-qPCR assay. To validate these findings, three of these five *penA* mutant strains that yielded amplification by conventional PCR were sequenced by Sanger; in all three, the presence of D345del and G545S confirmed the MAMA-qPCR results.

The amino acid sequences from codon 341 to codon 555 for *penA* gene analysis from 27 sequenced samples were also obtained. An adjustment of +1 has been applied to the positions starting from codon 345 to account for the insertion of D345ins at codon 346 in the non-mosaic *penA* alleles. Among the sequenced samples, all 27 sequenced samples carried the F504L and the A510V substitutions, 8 mosaic samples carried the G545S mutation, and notably all these samples also had an N512Y substitution. Additionally, 16/27 (59.3%) carried the D345ins at codon 346 of PBP-2, indicating the non-mosaic allele type. Of these non-mosaic samples, 12/16 (75.0%) were collected between 2019 and 2021; the last 11 samples (40.7%) displayed various substitutions compared to the wild type reference strain and lacked the D345ins, classifying them as mosaic types.

**Figure 1:**
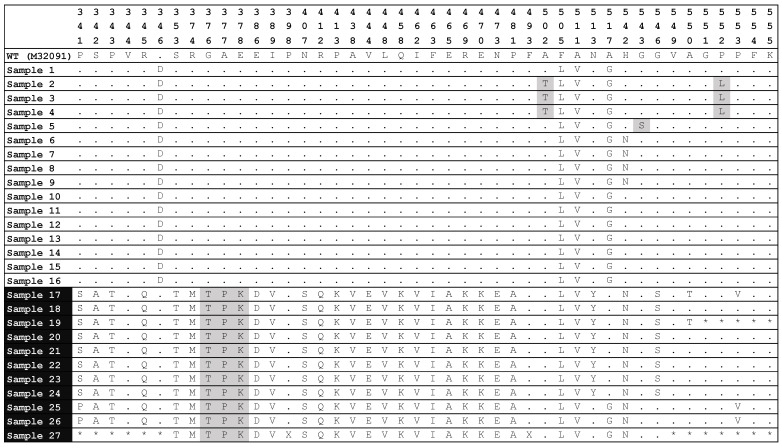
Amino acid variations in PBP-2 among the 27 successfully sequenced rectal clinical specimens of *N. gonorrhoeae*. The altered positions in the amino acid sequences were compared with the wild-type *N. gonorrhoeae* PBP-2 sequence (M32091). The residue numbering for wild type and mosaic variants (samples 17 to 27) was adjusted by +1 to ensure proper alignment with the non-mosaic alleles (samples 1 to 16 containing an aspartate insertion after position 345). Dots represent agreement with the wild-type sequence, while an amino acid letter represents a change in the codon position. The amino acids highlighted in grey represent important codon-level mutations that could confer decreased susceptibility to cefixime at positions 375–377, 501, 542, and 551. WT: Wild type.

With respect to the mutant codons related to decreased cefixime susceptibility, all the mosaic *penA* alleles detected carrying substitutions at codons 375–377, three of the non-mosaic alleles exhibited both A501T and P551L substitutions. Another notable finding was the G542S substitution, observed exclusively in one non-mosaic sample collected between 2019 and 2021. A total of 15/27 (55.6%) sequenced samples had at least one mutation at the 375–377, 501, 542, or 551 positions that can confer decreased susceptibility to cefixime (Figure 1).

## 4. Discussion

We collected *N. gonorrhoeae*-positive rectal clinical specimens from two periods (2013–2016 and 2019–2021) and analyzed them for azithromycin resistance mutations in the 23S rRNA gene and cephalosporins-decreased susceptibility mutations in the *penA* gene using MAMA-qPCR and Sanger sequencing. In our study, resistance-related mutations in the 23S rRNA and *penA* genes of *N. gonorrhoeae* positive rectal clinical specimens by NAATs were detected, identifying two samples carrying the A2059G mutation in the 23S rRNA gene, one of these carrying the mutation in all four alleles. Previous studies have demonstrated the strong association between the A2059G mutation and high-level azithromycin resistance; even the number of mutated alleles is directly and positively correlated with this high resistance [16,17,18]. Recent studies confirm this globally: Melendez et al. (2024) reported 89.9% azithromycin resistance in A2059G-positive strains [31], while Gianecini et al. (2023) documented this mutation in 13 *N. gonorrhoeae* strains with high-level azithromycin phenotypic results [13]. Our findings could indicate the presence of mutated strains circulating in our country, highlighting the need for continued surveillance and molecular monitoring.

Mutations in the *penA* gene that are linked to decreased susceptibility to cephalosporins were detected. Specifically, D345del indicates the presence of a mosaic *penA* allele, and the G545S mutation indicates a *penA* mosaic XXXIV variant in *N. gonorrhoeae*, a globally recognized recombinant allele that contains up to 68 amino acid substitutions, including A501V and G545S mutations [11]. These mutations have been previously observed in strains with reported resistance and decreased susceptibility to cephems and penicillin [10,21,32]. Mosaic *penA* alleles arise from extensive DNA recombination events between commensal and pathogenic *Neisseria* species, resulting in a high number of amino acid substitutions. These substitutions cause conformational changes at the active site of PBP-2 and reduce the antibiotic effectiveness [30,33]. Our findings reveal the presence of amino acid changes in circulating *N. gonorrhoeae* strains that may lead to clinical treatment failures with cephalosporins.

Our study identified three samples carrying the P551L mutation, which is significantly associated with decreased susceptibility to ceftriaxone [34]. These samples were classified as non-mosaic alleles, which also contained the A501T and F504L mutations. Prior studies have observed that the A501T/V substitution, in combination with specific non-mosaic alleles such as non-mosaic type IV and type IX, contributes significantly to decreased susceptibility to cephalosporins [20,35]. An association between F504L and P551S combined mutations has been found in penicillin-resistant strains [24]. However, in our results, the F504L was found in combination with the P551L mutation. Further research is required to comprehensively understand the impact of this specific combination on the PBP-2 structure and function in *N. gonorrhoeae* strains, particularly if this combination contributes to decreased susceptibility to cephalosporins.

In this study, mutations G375T, A376P, E377K, A501T, G542S, and P551L in the PBP-2 protein of *N. gonorrhoeae* were identified. These mutations are strongly related to decreased susceptibility to cefixime; it has been reported that any mutation at 375–377, 501, 542, or 551 codons can predict decreased cefixime susceptibility with an accuracy of 98.5% [22,25]. In particular, the A501T substitution has been identified in strains with decreased susceptibility to cephalosporins and is associated with a two-fold increase in ceftriaxone MICs. This mutation increases the rigidity of the active site, preventing the binding of cephalosporins to PBP-2 [36,37]. Furthermore, our findings underscore the potential for using these molecular markers to predict cephalosporin susceptibility.

The present study has some limitations. Only a small aliquot of each sample was available as these were remnants from the AC2 transport buffer, which releases and preserves RNA. This limited DNA quantity may have hindered detection in PCR assays, especially considering that these are clinical samples containing human DNA. Another limitation was the lack of information on resistance phenotypes in the processed samples, as no culture was performed at the time of collection, and only genetic material was collected for detection using molecular methods. Despite these limitations, our study provides clinically relevant data. Additional studies will be necessary for our country to reveal the association between these molecular resistance markers and the corresponding phenotype in other circulating *N. gonorrhoeae* strains.

To our knowledge, this is the first report from Peru of the A2059G mutation in the 23S rRNA gene detected in rectal clinical specimens positive for *N. gonorrhoeae*, highlighting the possibility of circulation and dissemination of these highly azithromycin-resistant strains, emphasizing the need to improve the epidemiological and molecular surveillance in *N. gonorrhoeae*. Additionally, our study detected strains carrying mutations associated with decreased susceptibility to ceftriaxone and cefixime, the antibiotics currently recommended for *N. gonorrhoeae* treatment. These findings underscore the importance of molecular monitoring to guide public health interventions in *N. gonorrhoeae* infections.

## 5. Conclusions

Our study was able to detect mutations related to high-level azithromycin resistance and decreased susceptibility to cephalosporins in clinical rectal specimens from patients with *N. gonorrhoeae* infection. We standardized and validated a MAMA-qPCR assay capable of detecting key resistance-associated mutations such as A2059G in the 23S rRNA gene and D345del/G545S in the *penA* gene, directly from rectal clinical specimens. This approach is valuable because *N. gonorrhoeae* does not always grow in culture, limiting conventional phenotypic testing; culture-independent identification offers a practical tool for routine clinical management and molecular surveillance of antimicrobial resistance in Peru. In addition, our results provide an overview of the circulating mutations conferring resistance to *N. gonorrhoeae* strains.

## Figures and Tables

**Table 1:** Primers used for the amplification of *N. gonorrhoeae* by MAMA-qPCR and sequencing, and their corresponding targets.

Primer Name	Sequence (5′ to 3′) ^1^	Product Size (bp) ^2^	Target (Antibiotic Affected) ^3^	Reference
C2611_F C2611_R	AACGTCGTGAGACAGTTTGGTTT GAACTTAGCTACCCGGCTATGCA	144	23S rRNA C2611T (moderate AZM resistance)	[26]
A2059_F A2059_R	TACAGTAAAGGTTCACGGGGTCAC TGGCCACACTGTCTCCTCCC	100	23S rRNA A2059G (high AZM resistance)	[26]
545_F 545_R	TGGTTAACGGTCGTTACGTCGATT GGCCCTGCCACTACACCGTT	142	Mosaic *penA* (decreased susceptibility/resistance to ESCs)	[26]
345_F 345_R	GGCAAAGTGGATGCAACCGAT GATAAACGTGGGTATCTTGTACGG	82	Mosaic *penA* (decreased susceptibility/resistance to ESCs)	[26]
gonrRNA-F gonrRNA-R2	ACGAATGGCGTAACGATGGCCACA TTCGTCCACTCCGGTCCTCTCGTA	712	23S rRNA sequencing	[16]
rRNA_1 rRNA_2 rRNA_3 rRNA_4	TCAGAATGCCACAGCTTACAAACT GCGACCATACCAAACACCCACAGG GATCCCGTTGCAGTGAAGAAAGTC AACAGACTTACTATCCCATTCAGC	2054 2240 2217 1847	23S rRNA sequencing (Alleles 1–4)	[16]
PenA-A3 PenA-B3	GCCGTAACCGATATGATCGA CGTTGATACTCGGATTAAGACG	862	*penA* sequencing	[30]

^1^ Non-template bases compared to the wild-type sequence of *N. gonorrhoeae* are underlined; ^2^ bp: base pair; ^3^ AZM: azithromycin; ESCs: extended-spectrum cephalosporins

**Table 2:** Proportion of mutant samples in the 23S rRNA and *penA* genes detected by the MAMA-qPCR assay. Total samples processed: N = 124 (23S rRNA gene), N = 86 (*penA* gene). A cut-off point was considered for C_T_ < 40 cycles.

Gene	Position Number (Nucleotide or Codon) ^1^	Wild Type Samples n (%)	Mutant Samples n (%) ^2^	Mutation Detected
23S rRNA	2611	42 (100.0%)	0 (0.0%)	C→T
	2059	62 (96.9%)	2 (3.1%)	A→G
*penA*	345	39 (88.6%)	5 (11.4%)	D345del
	545	58 (93.6%)	4 (6.4%)	G545S

^1^ Represents the nucleotide positions for the 23S rRNA gene and the codon positions for the *penA* gene. ^2^ The percentages of mutant samples were calculated based on the samples that did amplify.

## Data Availability

Data is unavailable due to privacy or ethical restrictions.

## References

[1] World Health Organization (2021). Global Progress Report on HIV, Viral Hepatitis and Sexually Transmitted Infections, 2021. Accountability for the Global Health Sector Strategies 2016–2021: Actions for Impact.

[2] Chan P.A., Robinette A., Montgomery M., Almonte A., Cu-Uvin S., Lonks J.R., Chapin K.C., Kojic E.M., Hardy E.J. (2016). Extragenital Infections Caused by Chlamydia Trachomatis and Neisseria Gonorrhoeae: A Review of the Literature. Infect. Dis. Obs. Gynecol..

[3] Szetela B., Łapiński Ł., Giniewicz K. (2023). Very High Incidence of Chlamydia Trachomatis, Neisseria Gonorrhoeae, and Treponema Pallidum among Low-Risk MSM in an Outpatient Clinic in Wroclaw, Poland in 2019–2020. Int. J. Environ. Res. Public Health.

[4] Minetti C., Rocha M., Duque L.M., Meireles P., Correia C., Cordeiro D., João I., Manita C., Soeiro S., Santos J.A. (2024). Orogenital and Anal Infection by Chlamydia Trachomatis, Neisseria Gonorrhoeae, Mycoplasma Genitalium, and Other Sexually Transmitted Infections in Men Who Have Sex with Men in Lisbon. Int. J. STD AIDS.

[5] Barbee L.A., Dombrowski J.C., Kerani R., Golden M.R. (2014). Effect of Nucleic Acid Amplification Testing on Detection of Extragenital Gonorrhea and Chlamydial Infections in Men Who Have Sex with Men Sexually Transmitted Disease Clinic Patients. Sex. Transm. Dis..

[6] Yang L.-G., Zhang X.-H., Zhao P.-Z., Chen Z.-Y., Ke W.-J., Ren X.-Q., Wang L.-Y., Chen W.-Y., Tucker J.D. (2018). Gonorrhea and Chlamydia Prevalence in Different Anatomical Sites among Men Who Have Sex with Men: A Cross-Sectional Study in Guangzhou, China. BMC Infect. Dis..

[7] Leon S.R., Segura E.R., Konda K.A., Flores J.A., Silva-Santisteban A., Galea J.T., Coates T.J., Klausner J.D., Caceres C.F. (2016). High Prevalence of Chlamydia Trachomatis and Neisseria Gonorrhoeae Infections in Anal and Pharyngeal Sites among a Community-Based Sample of Men Who Have Sex with Men and Transgender Women in Lima, Peru. BMJ Open.

[8] Unemo M., Shafer W.M. (2014). Antimicrobial Resistance in Neisseria Gonorrhoeae in the 21st Century: Past, Evolution, and Future. Clin. Microbiol. Rev..

[9] da Costa-Lourenço A.P.R., Barros Dos Santos K.T., Moreira B.M., Fracalanzza S.E.L., Bonelli R.R. (2017). Antimicrobial Resistance in Neisseria Gonorrhoeae: History, Molecular Mechanisms and Epidemiological Aspects of an Emerging Global Threat. Braz. J. Microbiol..

[10] Takahata S., Senju N., Osaki Y., Yoshida T., Ida T. (2006). Amino Acid Substitutions in Mosaic Penicillin-Binding Protein 2 Associated with Reduced Susceptibility to Cefixime in Clinical Isolates of Neisseria Gonorrhoeae. Antimicrob. Agents Chemother..

[11] Gose S., Nguyen D., Lowenberg D., Samuel M., Bauer H., Pandori M. (2013). Neisseria Gonorrhoeae and Extended-Spectrum Cephalosporins in California: Surveillance and Molecular Detection of Mosaic penA. BMC Infect. Dis..

[12] Zhao Y., Le W., Genco C.A., Rice P.A., Su X. (2023). Increase in Multidrug Resistant Neisseria Gonorrhoeae FC428-Like Isolates Harboring the Mosaic penA 60.001 Gene, in Nanjing, China (2017–2020). Infect. Drug Resist..

[13] Gianecini R.A., Poklepovich T., Golparian D., Cuenca N., Scocozza L., Bergese S., Canigia L.F., Vilches V., Lazzarino Elgart M.J., Unemo M. (2023). Sustained Transmission of Neisseria Gonorrhoeae Strains with High-Level Azithromycin Resistance (MIC ≥ 256 μg/mL) in Argentina, 2018 to 2022. Microbiol. Spectr..

[14] Jorge-Berrocal A., Vargas-Herrera N., Benites C., Salazar-Quispe F., Mayta-Barrios M., Barrios-Cárdenas Y.J., Melano R.G., Yagui M. (2022). Neisseria gonorrhoeae Surveillance Working Group Antimicrobial Susceptibility of Neisseria Gonorrhoeae Isolates from Peru, 2018 and 2019. Sex. Transm. Dis..

[15] Qquellon J., Vargas S.K., Eguiluz M., Vasquez F., Durand D., Allan-Blitz L.-T., Konda K.A., Ochoa T.J., Caceres C.F., Klausner J.D. (2023). Extra-Genital Neisseria Gonorrhoeae Infections with Genetic Mutations Conferring Ciprofloxacin Resistance among Men Who Have Sex with Men and Transgender Women in Lima, Peru. Int. J. STD AIDS.

[16] Ng L.-K., Martin I., Liu G., Bryden L. (2002). Mutation in 23S rRNA Associated with Macrolide Resistance in Neisseria Gonorrhoeae. Antimicrob. Agents Chemother..

[17] Zhang J., van der Veen S. (2019). Neisseria Gonorrhoeae 23S rRNA A2059G Mutation Is the Only Determinant Necessary for High-Level Azithromycin Resistance and Improves in Vivo Biological Fitness. J. Antimicrob. Chemother..

[18] Chisholm S.A., Dave J., Ison C.A. (2010). High-Level Azithromycin Resistance Occurs in Neisseria Gonorrhoeae as a Result of a Single Point Mutation in the 23S rRNA Genes. Antimicrob. Agents Chemother..

[19] Allen V.G., Mitterni L., Seah C., Rebbapragada A., Martin I.E., Lee C., Siebert H., Towns L., Melano R.G., Low D.E. (2013). Neisseria Gonorrhoeae Treatment Failure and Susceptibility to Cefixime in Toronto, Canada. JAMA.

[20] de Laat M.M., Wind C.M., Bruisten S.M., Dierdorp M., de Vries H.J., Schim van der Loeff M.F., van Dam A.P. (2019). Ceftriaxone Reduced Susceptible Neisseria gonorrhoeae in the Netherlands, 2009 to 2017: From PenA Mosaicism to A501T/V Nonmosaicism. Sex. Transm. Dis..

[21] Ameyama S., Onodera S., Takahata M., Minami S., Maki N., Endo K., Goto H., Suzuki H., Oishi Y. (2002). Mosaic-like Structure of Penicillin-Binding Protein 2 Gene (penA) in Clinical Isolates of Neisseria Gonorrhoeae with Reduced Susceptibility to Cefixime. Antimicrob. Agents Chemother..

[22] Deng X., Klausner J.D. (2020). Six penA Codons Accurately and Reliably Predict Cefixime-Decreased Susceptibility in Neisseria Gonorrhoeae. J. Infect. Dis..

[23] Brannigan J.A., Tirodimos I.A., Zhang Q.Y., Dowson C.G., Spratt B.G. (1990). Insertion of an Extra Amino Acid Is the Main Cause of the Low Affinity of Penicillin-Binding Protein 2 in Penicillin-Resistant Strains of Neisseria Gonorrhoeae. Mol. Microbiol..

[24] Powell A.J., Tomberg J., Deacon A.M., Nicholas R.A., Davies C. (2009). Crystal Structures of Penicillin-Binding Protein 2 from Penicillin-Susceptible and -Resistant Strains of Neisseria Gonorrhoeae Reveal an Unexpectedly Subtle Mechanism for Antibiotic Resistance. J. Biol. Chem..

[25] Deng X., Allan-Blitz L.-T., Klausner J.D. (2019). Using the Genetic Characteristics of Neisseria Gonorrhoeae Strains with Decreased Susceptibility to Cefixime to Develop a Molecular Assay to Predict Cefixime Susceptibility. Sex Health.

[26] Donà V., Smid J.H., Kasraian S., Egli-Gany D., Dost F., Imeri F., Unemo M., Low N., Endimiani A. (2018). Mismatch Amplification Mutation Assay-Based Real-Time PCR for Rapid Detection of Neisseria Gonorrhoeae and Antimicrobial Resistance Determinants in Clinical Specimens. J. Clin. Microbiol..

[27] Nadal-Baron P., Salmerón P., García J.N., Trejo-Zahinos J., Sulleiro E., Lopez L., Jiménez de Egea C., Zarzuela F., Ruiz E., Blanco-Grau A. (2022). Neisseria Gonorrhoeae Culture Growth Rates from Asymptomatic Individuals with a Positive Nucleic Acid Amplification Test. Lett. Appl. Microbiol..

[28] Kojima N., Park H., Konda K.A., Joseph Davey D.L., Bristow C.C., Brown B., Leon S.R., Vargas S.K., Calvo G.M., Caceres C.F. (2017). The PICASSO Cohort: Baseline Characteristics of a Cohort of Men Who Have Sex with Men and Male-to-Female Transgender Women at High Risk for Syphilis Infection in Lima, Peru. BMC Infect. Dis..

[29] Cordioli M., Gios L., Erbogasto A., Mirandola M., Sandri A., Padovese V., Caceres C., Vargas S., Blondeel K., Silva R. (2024). Clinic-Based Evaluation of the Dual Xpert CT/NG Assay on the GeneXpert System for Screening for Extragenital Chlamydial and Gonococcal Infections amongst Men Who Have Sex with Men. BMC Infect. Dis..

[30] Ito M., Deguchi T., Mizutani K.-S., Yasuda M., Yokoi S., Ito S.-I., Takahashi Y., Ishihara S., Kawamura Y., Ezaki T. (2005). Emergence and Spread of Neisseria Gonorrhoeae Clinical Isolates Harboring Mosaic-Like Structure of Penicillin-Binding Protein 2 in Central Japan. Antimicrob. Agents Chemother..

[31] Melendez J.H., Edwards V.L., Muniz Tirado A., Hardick J., Mehta A., Aluvathingal J., D’Mello A., Gaydos C.A., Manabe Y.C., Tettelin H. (2024). Local emergence and global evolution of Neisseria gonorrhoeae with high-level resistance to azithromycin. Antimicrob. Agents Chemother..

[32] Unemo M., Shafer W.M. (2011). Antibiotic Resistance in Neisseria Gonorrhoeae: Origin, Evolution, and Lessons Learned for the Future. Ann. N. Y. Acad. Sci..

[33] Bowler L.D., Zhang Q.Y., Riou J.Y., Spratt B.G. (1994). Interspecies Recombination between the penA Genes of Neisseria Meningitidis and Commensal Neisseria Species during the Emergence of Penicillin Resistance in N. Meningitidis: Natural Events and Laboratory Simulation. J. Bacteriol..

[34] Whiley D.M., Goire N., Lambert S.B., Ray S., Limnios E.A., Nissen M.D., Sloots T.P., Tapsall J.W. (2010). Reduced Susceptibility to Ceftriaxone in Neisseria Gonorrhoeae Is Associated with Mutations G542S, P551S and P551L in the Gonococcal Penicillin-Binding Protein 2. J. Antimicrob. Chemother..

[35] Bharat A., Demczuk W., Martin I., Mulvey M.R. (2015). Effect of Variants of Penicillin-Binding Protein 2 on Cephalosporin and Carbapenem Susceptibilities in Neisseria Gonorrhoeae. Antimicrob. Agents Chemother..

[36] Tomberg J., Fedarovich A., Vincent L.R., Jerse A.E., Unemo M., Davies C., Nicholas R.A. (2017). Alanine 501 Mutations in Penicillin-Binding Protein 2 from Neisseria Gonorrhoeae: Structure, Mechanism, and Effects on Cephalosporin Resistance and Biological Fitness. Biochemistry.

[37] Liao Y., Xie Q., Yin X., Li X., Xie J., Wu X., Tang S., Liu M., Zeng L., Pan Y. (2024). penA Profile of Neisseria Gonorrhoeae in Guangdong, China: Novel penA Alleles Are Related to Decreased Susceptibility to Ceftriaxone or Cefixime. Int. J. Antimicrob. Agents.

